# Research on Recognition Technology of Aluminum Profile Surface Defects Based on Deep Learning

**DOI:** 10.3390/ma12101681

**Published:** 2019-05-23

**Authors:** Ruofeng Wei, Yunbo Bi

**Affiliations:** 1 Key Laboratory of Advanced Manufacturing Technology of Zhejiang Province, College of Mechanical Engineering, Zhejiang University, Hangzhou 310027, China; wrfWRF1994@163.com; 2 State Key Laboratory of Fluid Power and Mechatronic System, College of Mechanical Engineering, Zhejiang University, Hangzhou 310027, China

**Keywords:** aluminum profile surface defects, multiscale defect-detection network, deep learning, average precision (AP), saliency maps

## Abstract

Aluminum profile surface defects can greatly affect the performance, safety, and reliability of products. Traditional human-based visual inspection has low accuracy and is time consuming, and machine vision-based methods depend on hand-crafted features that need to be carefully designed and lack robustness. To recognize the multiple types of defects with various size on aluminum profiles, a multiscale defect-detection network based on deep learning is proposed. Then, the network is trained and evaluated using aluminum profile surface defects images. Results show 84.6%, 48.5%, 96.9%, 97.9%, 96.9%, 42.5%, 47.2%, 100%, 100%, and 43.3% average precision (AP) for the 10 defect categories, respectively, with a mean AP of 75.8%, which illustrate the effectiveness of the network in aluminum profile surface defects detection. In addition, saliency maps also show the feasibility of the proposed network.

## 1. Introduction

Aluminum alloys have drawn more and more attention in aerospace engineering, automotive, and electronics industries due to their low density, high specific strength, good corrosion resistance, and good recycling ability [1,2]. Aluminum profile is an application form of aluminum alloys, and the demand for it is extremely large because of the massive use of space-frame constructions in high-speed rail and auto body [3]. Therefore, the surface quality of aluminum profiles has assumed significant importance. Any surface defects, such as cracks and deformations, will greatly affect the performance, safety, and reliability of products. Traditionally, human-based visual inspection is a common detection method in manufacturing engineering. However, due to low sampling rate, low precision, poor real-time performance, fatigue, greatly influenced by artificial experience, and other adverse factors, the artificial inspection is not sufficient to guarantee the stability and accuracy of detection. In addition, the other methods based on various signals, like electrical signal and magnetic signal, were also utilized to detect the surface defects by many companies. Asea Brown Boveri (ABB) Metallurgy [4] developed the Decraktor detection unit P1 using the principle of multi-frequency eddy current testing. The device can suppress the influence of various interference noises, thereby improving the reliability of the steel plate detection. However, eddy current testing can only detect conductors and needs to be close to the surface being inspected. Besides, the rough surface affects the detection result and the penetration depth of eddy current detector is limited.

Machine vision-based method for surface defect detection has an absolute advantage in terms of its safety, reliability, convenience, and efficiency. It is an effective means to realize the automation and intellectualization of the manufacturing processes in the steel and iron industry [5,6]. A typical machine vision-based defect-detection method consists of light source, Charge Coupled Device (CCD) camera, and image processing algorithms [7]. Many scholars have conducted significant research on image processing algorithms. In [8], Chondronasios A et al. used gradient-only co-occurrence matrices (GOCM) to classify two types of defects in an extruded aluminum profile. Besides, there is not much literature about aluminum profile defect detection using machine vision technology. Nevertheless, the problem can be seen as defect detection in metal material, such as steel and iron, which has been studied for many years in computer vision. 

Mathematical morphology is a technique of image analysis based on set theory, topology, and random functions. Dupont et al. [9] proposed a method using the cost matrix theory based on mathematical morphology, and the K-nearest neighbor (KNN) [10] classifier to detect eight kinds of defects on flat steel products. Spatial filtering is an image processing method that directly manipulates the pixels in an image. The gradient filters, like Sobel, Robert, Canny, and Laplacian filters are popular tools to detect points, lines, and edges in spatial filtering. Guo et al. [11] used the Sobel gradient operator and Fisher discriminant to detect defects on the steel surface. Moreover, Wu et al. [12] adopted a method based on fast Fourier transform (FFT) combined with a local border search algorithm for the recognition of hot-rolled steel strips. In [13], Yazdchi et al. applied a multifractal-based segmentation method to detach the region of defects from images, and then extracted ten features from the detected region for classification. A method in [14] using the Markov random field for texture analysis combined with a KNN classifier was used for classification of steel surface defects.

Although traditional machine vision-based methods using the CCD camera and image processing algorithms achieved the automatic detection of surface defects, the hand-designed features [15] used in the defect detection needed to be carefully designed by a programmer who well understands the domain of the task, which lacks robustness and is not conducive to the identification, classification, and detection of surface defects.

In recent years, due to the advances of artificial intelligence and deep learning, more concretely the convolutional neural network (CNN) [15,16], the quality of image classification, object detection, and face recognition have been rapidly developed. Deep learning which is based on artificial neural network discovers the distributed representation of its input data by transforming the data and low-level features into a more abstract and composite representation; the CNN can learn highly abstract and invariable features from large training datasets automatically, rather than constructing low-level features artificially; therefore, it can be robustly adapted to various computer vision tasks.

In 2012, as the winner of the ImageNet Large Scale Visual Recognition Challenge (ILSVRC) [17,18], Krizhevsky et al. [15] rekindled interests in CNN by firstly using deeper and wider networks in computer vison tasks. In [19], Girshick et al. proposed region-based CNN (R-CNN) which uses selective search [20] to generate around 2000 region proposals and “AlexNet” [15] to extract features, and a set of Support Vector Machines (SVMs) [21] and a regression model to classify and localize objects. However, the Region-CNN (R-CNN) is computationally expensive and slow, and not widely used in actual applications because it requires thousands of forward computations from the CNN to perform object detection for a single image. To address the drawbacks of R-CNN, Fast R-CNN [22] was developed by Girshick et al. Fast R-CNN only performs CNN forward computation on the image as a whole, so it shows higher speed and accuracy than R-CNN. Despite the better performance, Fast R-CNN generates many proposed regions through an external method like selective search which is time consuming. In 2016, a region proposal network (RPN) was presented to generate nearly cost-free region proposals in [23], and Ren et al. introduced the Faster R-CNN by combining the RPN and Fast R-CNN for object detection. The Faster R-CNN reduces the computational cost through sharing convolutional features between RPN and Fast R-CNN. Moreover, the novel RPN also improves the overall precision of object detection.

In computer vision, the objects of deep learning are often natural images, including pedestrians, vehicles, animals, and human faces, etc., but there are few studies on aluminum profile surface defects detection using the deep learning. Therefore, in this paper, we propose a multiscale defect-detection network for detecting the aluminum profile surface defects, which was based on the use of CNNs. The network is based on Faster R-CNN and the feature pyramid network (FPN) [24], and can effectively detect surface defects with various scales.

The rest of this paper is organized as follows. In Section 2, the dataset for training and evaluating the network is described. The multiscale defect-detection network is presented in Section 3, including the architecture of the network and how to train the network. Section 4 gives an experiment for training the network, including the implementation details of the experiment and the loss in the training process. In Section 5, the evaluation results of the network are presented. The paper ends with a summary of the major findings in Section 6. 

## 2. Dataset

### 2.1. Dataset of the Network

**Figure 1:**
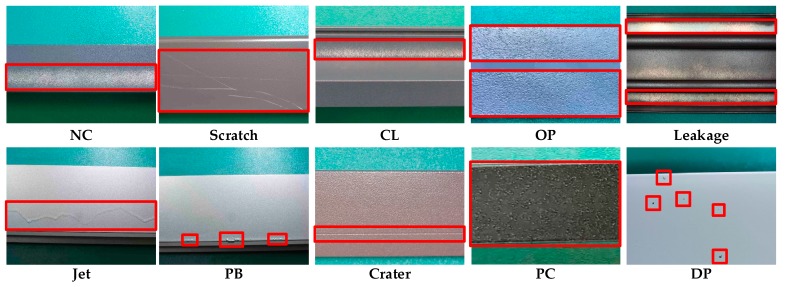
Images of aluminum profile surface defects.

**Figure 2:**
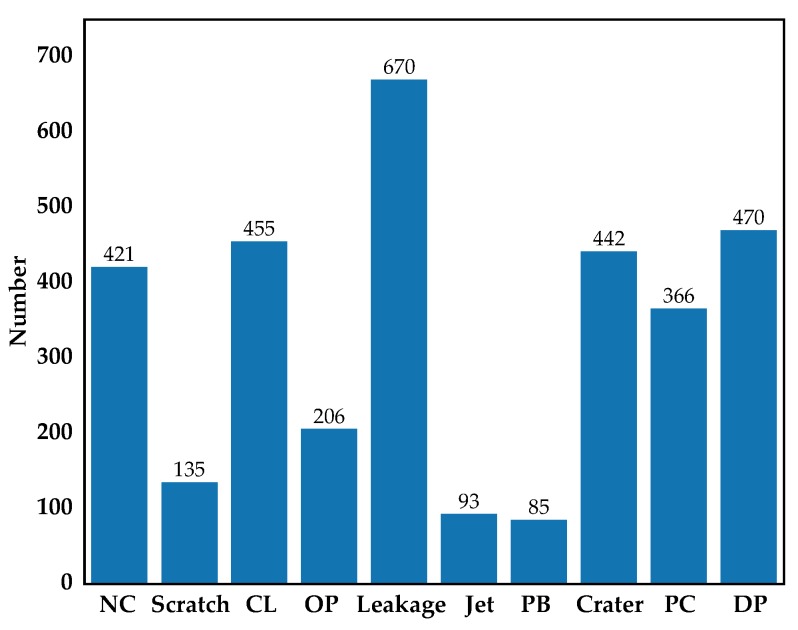
Diagram of the number of images with defects in each category.

Figure 1 shows the images of aluminum profile surface defects for training and evaluating the multiscale defect-detection network, and the resolution of each image was 2560 × 1920 pixels. The aluminum profile surface defects dataset was from [25]. There were ten types of defects on the aluminum profile: Non-Conductive (NC), Scratch, Corner Leak (CL), Orange Peel (OP), Leakage, Jet, Paint Bubble (PB), Crater, Parti-color (PC), Dirty Point (DP), and the defects were marked with a ground truth box which is like the red rectangle in Figure 1. The total number of images with defects was 3005, including 2776 images with a single type of defect and 229 images with multiple types of defects. To obtain the training dataset, images were randomly chosen from the overall dataset so that the training dataset contained about 90% of images of each defect type. Thus, there were 2705 images for training and 300 images for testing. Figure 2 shows the number of images with defects in each category.

As can be seen in Figure 1, some defects were particularly small, such as PB and DP, and some defects were extremely narrow and long, like Crater. The defects with abnormal size increased the difficulty of defect detection.

### 2.2. Data Augmentation

Convolutional neural networks (CNNs), especially deep ones, are likely to be prone to overfitting when the training dataset is small. The aluminum profile surface defects dataset we used comprised only several thousand samples, which may not fully sustain the training of deep CNNs. Moreover, it is time-consuming and laborious to make large training dataset with defect location annotations by professional annotators.

**Figure 3:**
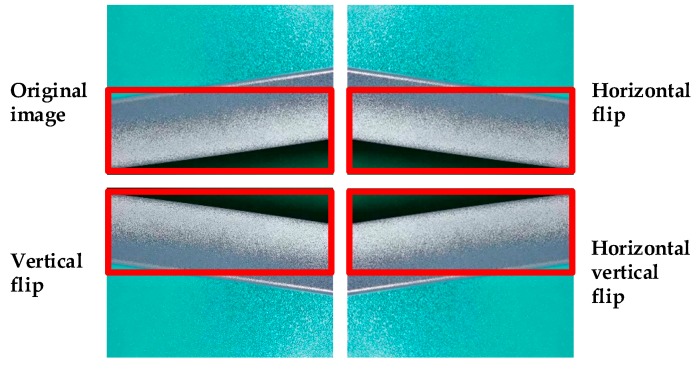
Data augmentation.

Therefore, a data augmentation technique [26] is performed over the original defect dataset with the aim of dataset expansion. To do so, each image and corresponding ground truth boxes are passed through some transformations: vertical flip, horizontal flip, and horizontal vertical flip (shown in Figure 3).

## 3. Method

**Figure 4:**
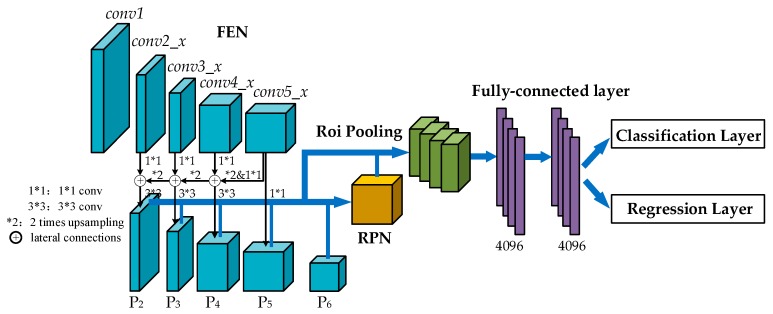
Architecture of the multiscale defect-detection network.

To classify and localize defects on the aluminum profile surface, the multiscale defect-detection network based on Faster R-CNN was proposed. The overall schematic architecture of the network is presented in Figure 4. The Faster R-CNN system was composed of Feature Extraction Network (FEN), Region Proposal Network (RPN), Region-of-Interesting (ROI) Pooling, and Classification and Regression Layer. Considering the characteristics of surface defects of aluminum profiles, the idea of feature fusion from FPN was added to the basic Faster R-CNN to improve defect detection performances. The details of the multiscale defect detection are explained in this section.

### 3.1. Feature Extraction Network 

**Figure 5:**
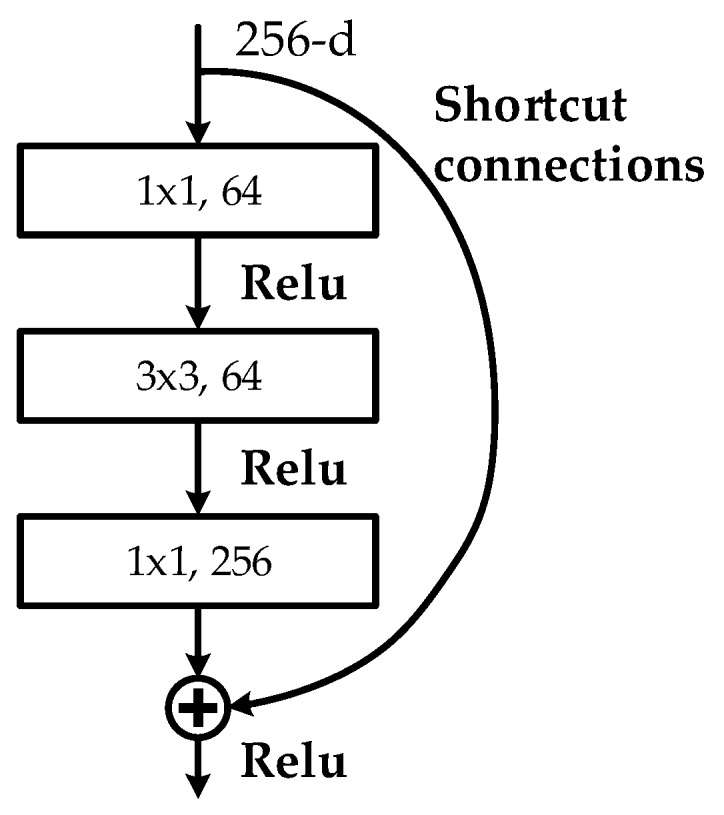
Structure of the *bottleneck*.

FEN is a large CNN that can automatically extract high-level features from input images. In this study, we used ResNet101 [27] to obtain high-level and semantically strong features. The basic structure of the ResNet101 is a *bottleneck* which solves the network performance degradation problem and leads the CNN model deeper than ever. As depicted in Figure 5, the *bottleneck* contains three convolutional layers: 1 × 1, 3 × 3, and 1 × 1 convolutional layers, which is followed by a “Relu” activation function [28], respectively, and “shortcut connections,” which are those skipping one or more layers and map input directly to output without adding extra parameters.

The detailed architecture of the ResNet101 for ImageNet is summarized in Table 1. The network was composed of *conv1*, *pool*, *conv2_x*, *conv3_x*, *conv4_x*, *conv5_x*, average pool, 1000 d full-connected layer (1000 d fc). The output of the fully connected layer was fed to a 1000-way softmax which could produce a probability distribution over 1000 classes. When applied to extract feature maps, we only use the *conv1*, *pool*, *conv2_x*, *conv3_x*, *conv4_x,* and *conv5_x*. The *conv2_x*, *conv3_x*, *conv4_x,* and *conv5_x* were constructed by *bottlenecks* stacked upon each other, and the number of *bottlenecks* of each section is shown in Table 1. Because of the “very deep” network, high-level semantic features that facilitate subsequent recognition could be obtained from the *conv5_x*. 

**Figure 6:**
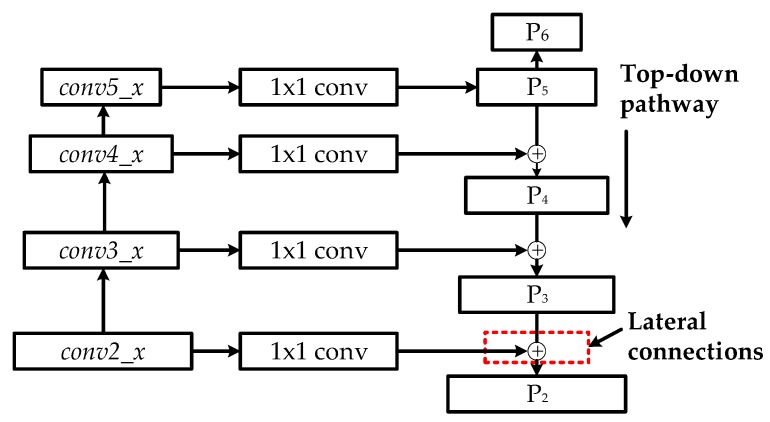
Architecture of feature fusion in the multiscale defect-detection network.

However, the high-level feature maps are usually low-resolution; so when small scale defects on the aluminum profile were mapped into high-level features, the representational capacity for the detection of these defects was weakened. The idea of feature fusion is to combine low-resolution, semantically-strong features with high-resolution, low-level features. As shown in Figure 6, the architecture of feature fusion in the multiscale defect-detection network adopted a top-down pathway and lateral connections. The top-down pathway produced higher resolution and semantically stronger features by up-sampling semantically stronger, but lower resolution feature maps to nearest lower level features’ scale, and then these features were added to the nearby low-level features via lateral connections. Therefore, a set of multiscale feature maps: {P_2_, P_3_, P_4_, P_5_} in which all levels are semantically strong were generated. In addition, an extra feature map P_6_, which is a simple two-stride subsampling of P_5_, was added to the output feature maps. It is worth noting that the feature maps {P_2_, P_3_, P_4_, P_5_, P_6_} would be transmitted to RPN and only {P_2_, P_3_, P_4_, P_5_} would be input into ROI pooling.

### 3.2. Region Proposal Network

**Figure 7:**
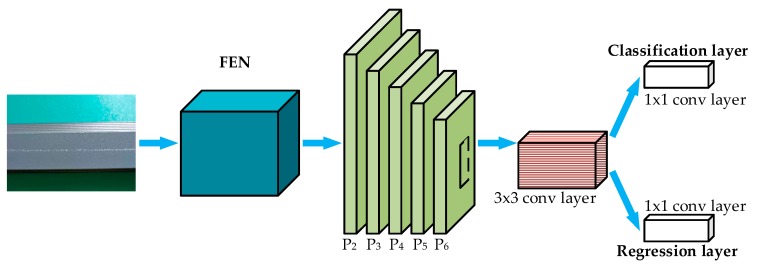
Structure of the improved RPN.

After feature fusing, RPN can generate region proposals or regions of interest (ROI), which are rectangular regions surrounding defects, including the probability of being foreground (containing defects) in each proposal. The schematic structure of an improved RPN is presented in Figure 7. The improved RPN is a fully convolutional network, which is naturally implemented with a 3 × 3 convolutional (conv) layer followed by two sibling 1 × 1 convolutional (conv) layers for classification and regression. Concretely, we attach the fully convolutional network (3 × 3 conv and two 1 × 1 convs) to each feature map output by FEN, and then several vectors containing estimate probability of defect/not-defect for each anchor and prediction coordinate transformation from anchors to region proposals are generated. 

**Figure 8:**
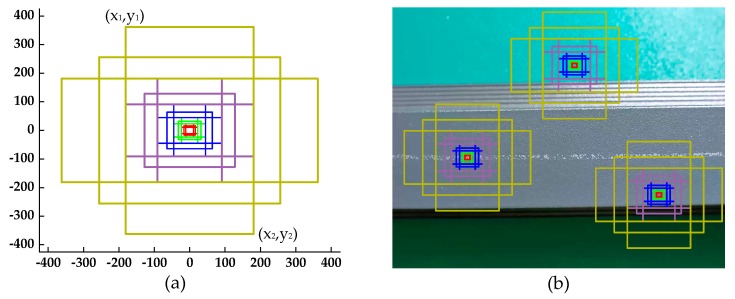
(**a**) Fifteen kinds of anchors; (**b**) anchors set on the input image.

Anchors play an important role in the improved RPN. An anchor is a reference box, determined by upper left and lower right coordinates: (x_1_, y_1_) and (x_2_, y_2_), as shown in Figure 8a. The anchor which is previously assigned on the input image transforms the defect detection problem into whether the anchor surrounds any defects and how far away the defect is from the anchor (shown in Figure 8b). Based on the work of Lin et al. [24], we defined the anchors to have areas of {32^2^, 64^2^, 128^2^, 256^2^, 512^2^} pixels corresponding to {P_2_, P_3_, P_4_, P_5_, P_6_} in the improved RPN. Moreover, the anchor in each feature map has three aspect ratios: {1:2, 1:1, 2:1}. In the improved RPN, there are 15 kinds of anchors, and approximately 306,900 anchors set on the input image. 

**Figure 9:**
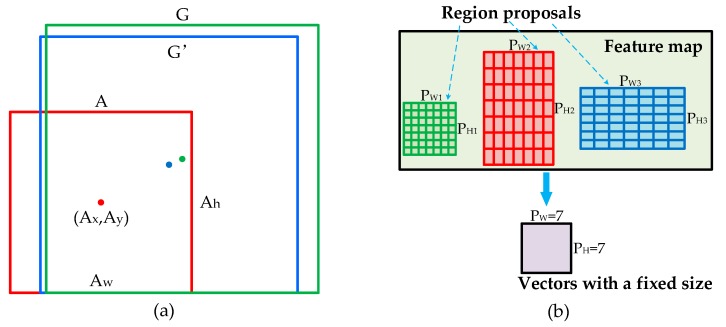
(**a**) The schematic diagram of the regression layer; (**b**) ROI pooling.

Although the improved RPN could create a large number of anchors, some of which may be close to defects, these anchors provide coarse localization and need to be refined. The regression layer is a simple, inexpensive technique which can compensate for the anchors’ weakness at localization. Concretely, it attempts to learn a transformation d × (A) that maps the anchors to the region proposal. As described in Figure 9a, A is the anchor, G is the ground truth box, and G’ is the predicting region proposal, which are specified as a set of center coordinates and a width and height in pixels, where A = (A_x_, A_y_, A_w_, A_h_). Thus, the transformation d × (A) could transform A into G’ which is closer to G and better captures the defect:

(1) $$\begin{array}{l} G^{\prime }{}_{x}=A_{w}d_{x}(A)+A_{x}\\ G^{\prime }{}_{y}=A_{h}d_{y}(A)+A_{y}\\ G^{\prime }{}_{w}=A_{w}\exp(d_{w}(A))\\ G^{\prime }{}_{y}=A_{h}\exp(d_{h}(A)) \end{array}$$

### 3.3. ROI Pooling

The region proposals output from the improved RPN have different dimensions, and the input of final classification and regression layer need to be the same size, so the purpose of ROI pooling is to perform max pooling to convert the features inside any proposals into vectors with a fixed size (e.g., 7 × 7). The specific operation of ROI pooling is shown in Figure 9b. Firstly, the region proposals with different sizes are divided into equal-sized sections, such as 7 × 7; then, the max value in each section is output, and fixed-size vectors can be obtained.

In addition, the region proposal with (x_1_, y_1_) and (x_2_, y_2_) need to be mapped to the feature maps before the operation ROI pooling. There are four feature maps {P_2_, P_3_, P_4_, P_5_} input into ROI pooling, so it is important to determine which feature map the region proposal belongs to. As per Lin [24], we assigned a region proposal of width (w) and height (h) (on the input image) to the feature map P_k_ by:

(2) $$k=4+\log_{2}(\sqrt{wh}/224)$$

Intuitively, if the region proposal’s scale is 512 × 512, it should be mapped to P_5_. The mapping method is to reduce coordinates of the anchor to the down-sampling multiple of the input image to the feature map. For example, we defined the anchor with area of 512^2^ pixels on the P_5_ feature map. Therefore, one anchor which is center at the input image with scale {−256, 256, 256, −256 } is {−8, 8, 8, −8} when mapped to P_5_.

### 3.4. Classification and Regression Layers

The classification and regression layers are composed of fully-connected layers. For the classification layer, it outputs a vector with the predicting probability of 11 classes (10 defects plus 1 background class); the regression layer outputs four parameters for each class to refine the region proposals again. Before the final classification and regression layer, there are two hidden, 1024 d fully connected layers which map the learned features to the sample space for classification and regression.

### 3.5. Network Training

The multiscale defect-detection network is composed of the architecture of the network and weights of the convolutional layers. When the design of the network structure was completed, we needed to obtain the optimal weights of the convolutional layers. Network training is a process that realizes the optimization of weights and leads the prediction of the network approximating to the truth of inputs, and it consists of forward propagation and backward propagation. Forward propagation is the calculation and storage of intermediate variables (including outputs) for the network in the order from input to output. Backward propagation refers to the method of calculating the losses (the difference between outputs and the truth of inputs) of the network and updating the weights using the gradient from the losses. The losses of the multiscale defect-detection network are from the improved RPN and the Classification and Regression layers. In the training, the selection of the losses is extremely significant.

The improved RPN is trained end-to-end, for both the classification and regression layers. We used the multitask loss L in Fast R-CNN [22] to train the improved RPN:

(3) $$L(p_{i},\;t_{i})=\frac{1}{N_{cls}}\sum L_{cls}(p_{i},\;p_{i}{}^{*})+\lambda \frac{1}{N_{reg}}\sum_{i}p_{i}{}^{*}\times L_{reg}(t_{i},\;t_{i}{}^{*})$$

where i is the index of an anchor in a mini-batch, and in the classification loss p* and p are the ground truth label and predicted probability of being defects in the anchor, respectively. In the regression loss, t_i_ and t_i_* are vectors representing the geometrical difference between the anchor and the predicting region proposal, as well as the anchor and the ground truth box, respectively, and t_i_* is calculated as:

(4) $$\begin{array}{l} t_{x}{}^{*}=(G_{x}-A_{x})/A_{w}\\ t_{y}{}^{*}=(G_{y}-A_{y})/A_{h}\\ t_{w}{}^{*}=\log(G_{w}/A_{w})\\ t_{h}{}^{*}=\log(G_{h}/A_{h}) \end{array}$$

Additionally, the classification loss is calculated as:

(5) $$L_{cls}=\sum_{i}-p_{i}{}^{*}\times \log(p_{i})-(1-p_{i}{}^{*})\times \log(1-p_{i})$$

The regression loss is calculated as:

(6) $$\begin{array}{l} L_{reg}=\sum_{i}smooth_{L1}(t_{i}{}^{*}-t_{i})\\ \left(smooth_{L1}(x)=\left\{ \begin{array}{l} 0.5x^{2}\;\mathrm{if}\;\left|x\right|<1\\ \left|x\right|-0.5\;\mathrm{otherwise} \end{array}\right.\right) \end{array}$$

Moreover, we use the same loss as the improved RPN to train the Classification and Regression layers, which are also trained end-to-end.

## 4. Experiments 

### 4.1. Implementation Details

In the research on recognition of aluminum profile surface defects, all experiments were performed using Python 3.5, PyTorch as the deep learning library, cuda 9.1 and cudnn 5.1 on Google Cloud Platform with a 8 GB memory NVIDA Tesla K80 graphics processing unit (GPU). While training the multiscale defect-detection network, we applied scaling of 960 on the shorter side of input images (with the resolution of 2560 × 1280), and then normalized each image by making it have fixed means and variances. When calculating the losses of the network, λ=1 was used both in RPN’s and in the Classification and Regression layers’ loss function. In addition, weights of the network were improved by stochastic gradient descent (SGD), including a learning rate of 0.001, momentum of 0.9, and weight decay of 0.0005. In the training of the network, we proposed the use of one in batch size (batch means the number of input images), and 50 epochs to improve the performance (epoch means the number of the network is trained). 

When evaluating the performance of an object detection network, average precision (AP) is often used. Detailed descriptions of AP can be seen in the paper by Everingham et al. [29]. In the multiscale defect detection, we used mean AP (mAP) which is defined as the average of calculated APs for ten types of defects to evaluate. 

### 4.2. Loss in Network Training

During the process of network training, it is necessary to visualize the loss on the training dataset in time. Whether the defect-detection network is effective can be judged by the trend of the loss curve. Moreover, the loss can guide the adjustment of parameters in the network, including training epoch, learning rate, weight decay, and structural optimization. The total losses of the multiscale defect-detection network consist of the classification loss and regression loss in RPN, and classification loss and location loss in the Classification and Regression layers. In the training, the various losses can be represented by total_loss, rpn_cls_loss, rpn_box_loss, cls_loss, and loc_loss, respectively.

**Figure 10:**
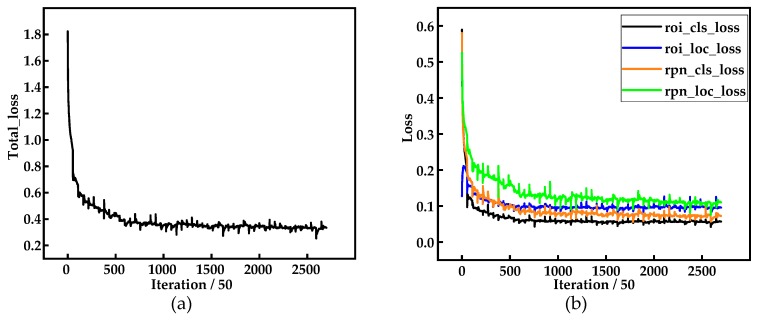
(**a**) The total_loss curve during the process of network training; and (**b**) the roi_cls_loss, roi_loc_loss, rpn_cls_loss, and rpn_loc_loss curves during the training.

The loss curves during the training are shown in Figure 10. In this study, the network is trained on all training dataset in each epoch, and the various losses of the network are recorded every 50 steps (one step equals one image), so there are 2700 iterations totally in all loss curves (one iteration equals 50 steps). As shown in Figure 10, all kinds of loss curves display a downward trend, and the loss decreases greatly at the beginning of training, indicating that the learning rate is appropriate and the gradient descent is carried out. Then, the loss curves tend to be stable after training to a certain epoch, which indicates that the network starts to converge. Thus, the parameters we selected in Section 4.1 are appropriate. 

## 5. Results and Discussion

After finishing the training of the network, we used 300 test images over 10 defect categories, randomly chosen from the overall dataset, to evaluate the multiscale defect-detection network. We recorded the AP for the ten types of defects and the mAP. Then, aluminum surface defect detection results output from the network were displayed. Lastly, we computed several saliency maps [30], specific to given images and classes, to represent the importance to the network at every location on input images.

### 5.1. mAP

Table 2 shows the mAP of Faster R-CNN and the multiscale defect-detection network at test images. For Faster R-CNN, mAP was 63.3%, and the detection time was 0.73 s per image. For the multiscale defect-detection network, we achieved a mAP of 75.8%, and the large improvement of the network over Faster R-CNN illustrates that the idea of feature fusion promotes recognition accuracy on aluminum profile surface defects. Additionally, the detection time of the network was a little more than Faster R-CNN, but the increase was not obvious and did not influence the defect detection. 

**Figure 11:**
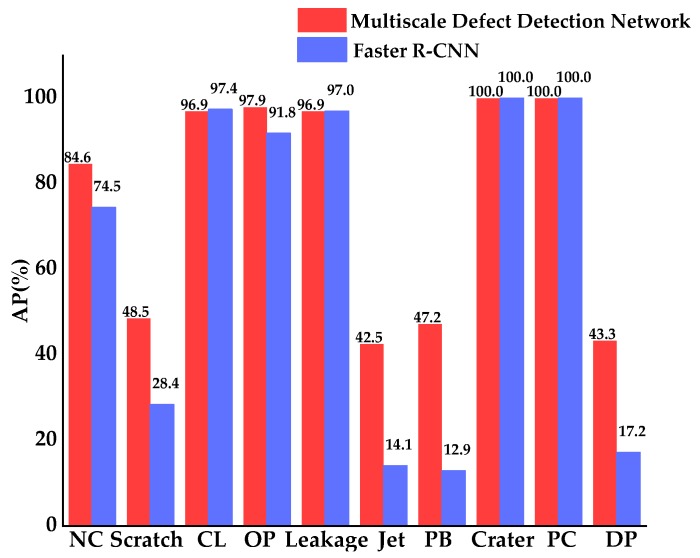
Average precisions (APs) for ten types of defects of Faster R-CNN and the multiscale defect-detection network.

The APs for ten types of defects of the Faster R-CNN and multiscale defect-detection network are shown in Figure 11. For Corner Leak (CL), Orange Peel (OP), Leakage, Crater, and Parti-Color (PC), both networks achieved high APs, nearly at 100%. For Paint Bubble (PB) and Dirty Point (DP), the scales of which were small, APs of the multiscale defect-detection network was greatly higher than Faster R-CNN, which illustrates the improvement of the network in the detection of small size defects. As can be seen in Table 2 and Figure 11, the mAP of the multiscale defect-detection network and the APs for several types of defects were still low, so there were many defects that were not detected. These missed detections may have been caused by the small training dataset. Therefore, in future studies, a larger dataset in each category will be generated to improve the network’s detection capacity.

### 5.2. Aluminum Surface Defect Detection Results

**Figure 12:**
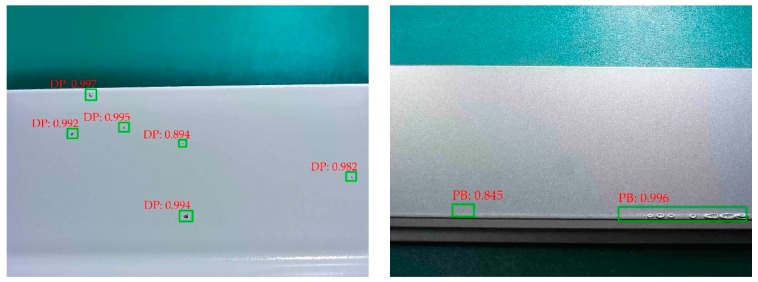

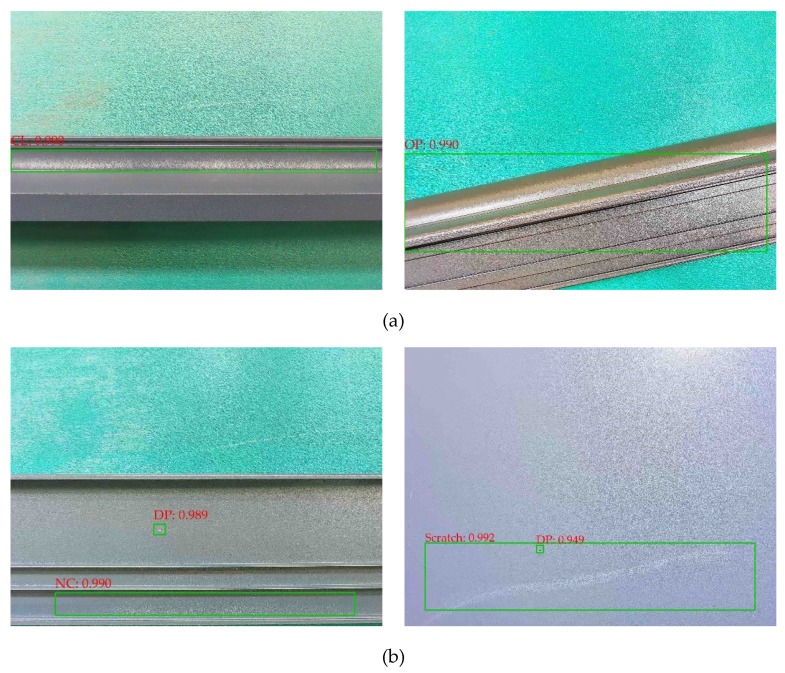
(**a**) Images with a single type of defect; (**b**) images with multiple types of defects.

Figure 12 shows the detection results of aluminum surface defect images using the multiscale defect-detection network. The position of the defects is marked by a green rectangular box, and the defect category and the confidence of the category are given in the upper left corner of the box.

Images with a single type of defect are shown in Figure 12a, DP, PB, CL, and OP are successfully detected. Besides, although DP scatters on the aluminum profile surface in Figure 12a, almost all of them are detected, which indicates that the network has a strong detection capability in small size defects. In Figure 12b, we display the images with multiple types of defects. Despite all defects being successfully recognized, the location of the rectangular boxes had some minor errors; for example, the box surrounding Non-Conducting (NC) was too large, and the box positioning the scratch did not completely surround the scratch. These problems are related to incorrect location annotations of aluminum surface defect images. 

### 5.3. Saliency Maps

A saliency map is an image that shows which pixels in the input image should be changed to affect the class score the most. Such pixels also are related to the defect location in the input image. Therefore, through the saliency map, it can intuitively analyze which part of the aluminum profile defect image that the multiscale defect-detection network is interested in and thus can verify the effectiveness of the network. The saliency map is computed by the derivative of the loss of the classification layer with respect to the input image in the backward propagation. Detailed calculation of the saliency map can be seen in the paper by Simonyan et al. [30]. 

**Figure 13:**
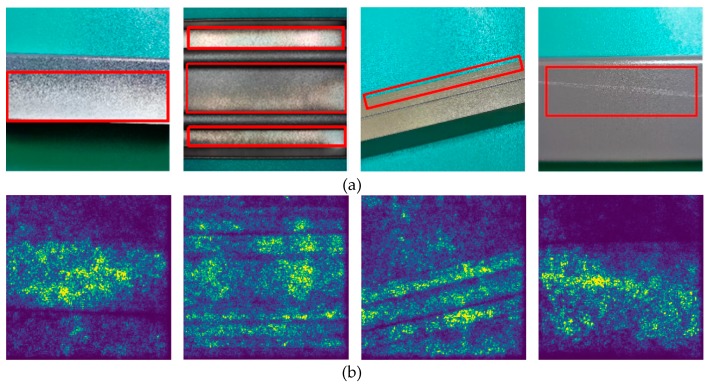
(**a**) Aluminum profile defect images; (**b**) saliency maps.

Figure 13 includes aluminum profile defect images and corresponding saliency maps. The defect images are shown in Figure 13a, and red rectangular boxes roughly indicate the location of the defects. In Figure 13b, we show the saliency maps in which the relatively bright place is the area where the multiscale defect-detection network focuses on. As can be seen in the figure, the focus of the network is on the aluminum profile, and the image background is selectively ignored. In addition, the brightness of the defect on the aluminum profile is particularly high in the saliency maps, indicating that the network mainly pays attention to the defects, which also confirms the effectiveness of the network.

## 6. Conclusions

Defects on aluminum profile surfaces will significantly influence the performance, reliability, and safety of products. Artificial inspection has low precision and is time consuming. Moreover, traditional machine vision-based defect detection methods overly rely on hand-crafted features, which lack robustness and need to be carefully designed by experienced programmers. Therefore, in this paper, we propose a defect-detection network based on deep learning which can automatically extract abstract invariable features from a large dataset, rather than designing low-level features artificially. The major findings of this paper can be summarized as follows:

(1) A multiscale defect-detection network based on Faster R-CNN is proposed to recognize aluminum profile surface defects. Considering the characteristics of defects on the aluminum profile surface, we add the idea of feature fusion to the basic Faster R-CNN to improve detection performances.
(2) The training images of aluminum profile surface defects are used to train the network. We adopt a data augmentation technique to expand the dataset. In the training process, we utilize the various losses to adjust parameters of the network.
(3) The performance of the trained multiscale defect-detection network is evaluated on 300 test images. Compared with Faster R-CNN, the multiscale defect-detection network achieves a higher mAP, which is 75.8%. For the small size defects (e.g., Paint Bubble and Dirty Point), the network gets higher APs, which illustrates the effectiveness of the feature fusion. In addition, we display the detection results of aluminum profile surface defects images and the saliency maps, which can verify the effectiveness of the network architecture.

In the future, more aluminum profile surface defects images in each category will be provided to train the network. In-depth analysis for the poor performance of the detection network in some types of defects will be performed, and the architecture will be improved.

## Figures and Tables

**Table 1:** Architecture of the ResNet101.

Layer Name	Output Size	101-Layer
conv1	112×112	7 × 7, 64, stride 2
pool	56×56	3 × 3 max pool, stride 2
conv2_x	56×56	$\left[\begin{array}{l} 1\times 1,\;64\\ 3\times 3,\;64\\ 1\times 1,\;256 \end{array}\right]\times 3$
conv3_x	28×28	$\left[\begin{array}{l} 1\times 1,\;128\\ 3\times 3,\;128\\ 1\times 1,\;512 \end{array}\right]\times 4$
conv4_x	14×14	$\left[\begin{array}{l} 1\times 1,\;256\\ 3\times 3,\;256\\ 1\times 1,\;1024 \end{array}\right]\times 23$
conv5_x	7×7	$\left[\begin{array}{l} 1\times 1,\;512\\ 3\times 3,\;512\\ 1\times 1,\;2048 \end{array}\right]\times 3$
	1×1	average pool, 1000 d fc, softmax

**Table 2:** Performance comparison with different networks.

Network	mAP (%)	Time (s/image)
**Faster Region- CNN (R-CNN)**	63.3	0.73
**Multiscale Defect-Detection Network**	75.8	0.84
